# Closing the Loop of Satellite Soil Moisture Estimation via Scale Invariance of Hydrologic Simulations

**DOI:** 10.1038/s41598-019-52650-3

**Published:** 2019-11-06

**Authors:** Giuseppe Mascaro, Ara Ko, Enrique R. Vivoni

**Affiliations:** 1 School of Sustainable Engineering and the Built Environment, Arizona State University, Tempe, AZ USA; 2 School of Earth and Space Exploration, Arizona State University, Tempe, AZ USA

**Keywords:** Hydrology, Hydrology

## Abstract

Surface soil moisture plays a crucial role on the terrestrial water, energy, and carbon cycles. Characterizing its variability in space and time is critical to increase our capability to forecast extreme weather events, manage water resources, and optimize agricultural practices. Global estimates of surface soil moisture are provided by satellite sensors, but at coarse spatial resolutions. Here, we show that the resolution of satellite soil moisture products can be increased to scales representative of ground measurements by reproducing the scale invariance properties of soil moisture derived from hydrologic simulations at hyperresolutions of less than 100 m. Specifically, we find that surface soil moisture is scale invariant over regimes extending from a satellite footprint to 100 m. We use this evidence to calibrate a statistical downscaling algorithm that reproduces the scale invariance properties of soil moisture and test the approach against 1-km aircraft remote sensing products and through comparisons of downscaled satellite products to ground observations. We demonstrate that hyperresolution hydrologic models can close the loop of satellite soil moisture downscaling for local applications such as agricultural irrigation, flood event prediction, and drought and fire management.

## Introduction

Water stored in the surface (0–5 cm) soil layer, referred to as surface soil moisture (SM), plays a key role in the climate system through its nonlinear feedbacks to energy, water and carbon fluxes^1,2^. SM is characterized by complex dynamics across a wide range of spatial and temporal scales^3^ that impact plant photosynthesis, streamflow, large-scale weather circulation, and, in the long term, global climate variability^1,4,5^. As a result, characterizing the spatiotemporal variability of SM is critical to support many practical applications, such as weather^6^ and flood^7^ forecasting, drought monitoring^8^, irrigation scheduling^9^, and climate prediction^10^. SM is routinely monitored through networks of ground stations. While useful and accurate, these observations are sparse and discontinuous. Satellite-borne microwave remote sensors are able to overcome this limitation, providing global estimates of surface SM every 1–3 days at resolutions of 25–50 km depending on sensor type and wavelength^11^. At these resolutions, satellite SM estimates can be directly used for hydroclimatological applications at continental and global scales. Unfortunately, their utility is limited to support local and regional hydrometeorological and agricultural applications that require information on SM variability at scales of 1 km or less.

Previous studies have shown that the resolution of satellite SM products can be increased through the use of statistical downscaling algorithms that reproduce scale invariant properties of SM^12–16^. Scale invariance is a property exhibited by numerous geophysical variables^17^. It emerges when a power-law relation links a metric characterizing the process of interest at a given scale (for example, the moments of its empirical distribution) with the scale. While simple, this relation has significant implications. It suggests that mechanisms that control a phenomenon at different scales have a deep connection, even if they are apparently unassociated^18^. In addition, it permits deriving information at scales that cannot be observed through the information available at other scales. Scale invariance has indeed been identified in SM fields in the regime from ~30 km (the satellite footprint scale) to ~1 km by analyzing products from airborne sensors in United States and Australia^12–16^. While promising, these results have been only obtained at a limited number of campaign sites and measurement periods, thus requiring further validation.

An alternative source of high-resolution SM estimates to investigate scale invariance are simulations from distributed hydrologic models. The potential for this was demonstrated through high-resolution simulations of two hydrologic models in a small (611 km^2^) basin in central United States, where model-derived SM data were shown to exhibit the same scale invariance properties of aircraft products^19,20^, and in a larger region using simulations at ~12-km resolution with a land surface model^21^. At the same time, progress has been made towards the feasibility of hydrologic simulations over large domains at hyperresolutions of *O*(100 m)^22–26^. This scale has been suggested as the upper limit at which the application of point representations of hydrologic processes is still physically meaningful^23^. If properly parameterized and tested, hyperresolutions simulations capture the spatial variability of hydrologic variables with unprecedented details. Their outputs provide unique data sets for expanding the use of SM scale invariance over larger regimes and to regional and continental areas.

## Results

### Scale invariance of hyperresolution soil moisture estimates

We use long-term hyperresolution hydrologic simulations over a large domain to show evidence of spatial scale invariance of SM from the satellite footprint scale to a representative area of ground observations. We then utilize this evidence to downscale satellite products. To do this, we use hydrologic simulations of SM at the hyperresolution of ~88 m with a physics-based distributed hydrologic model^27–29^ in the Río Sonora basin (RSB), Mexico^30^. Simulations are conducted from 2004 to 2013 and thoroughly tested against SM observations from a network of ground stations and remotely-sensed estimates of land surface temperature (see Methods). Since the hydrologic model operates on an irregular mesh, outputs are resampled into a regular grid at 125-m. To date, this represents the most extensive hyperresolution data sets of surface SM in a real-world basin in terms of duration (a decade) and domain size (21,237 km^2^).

**Figure 1:**
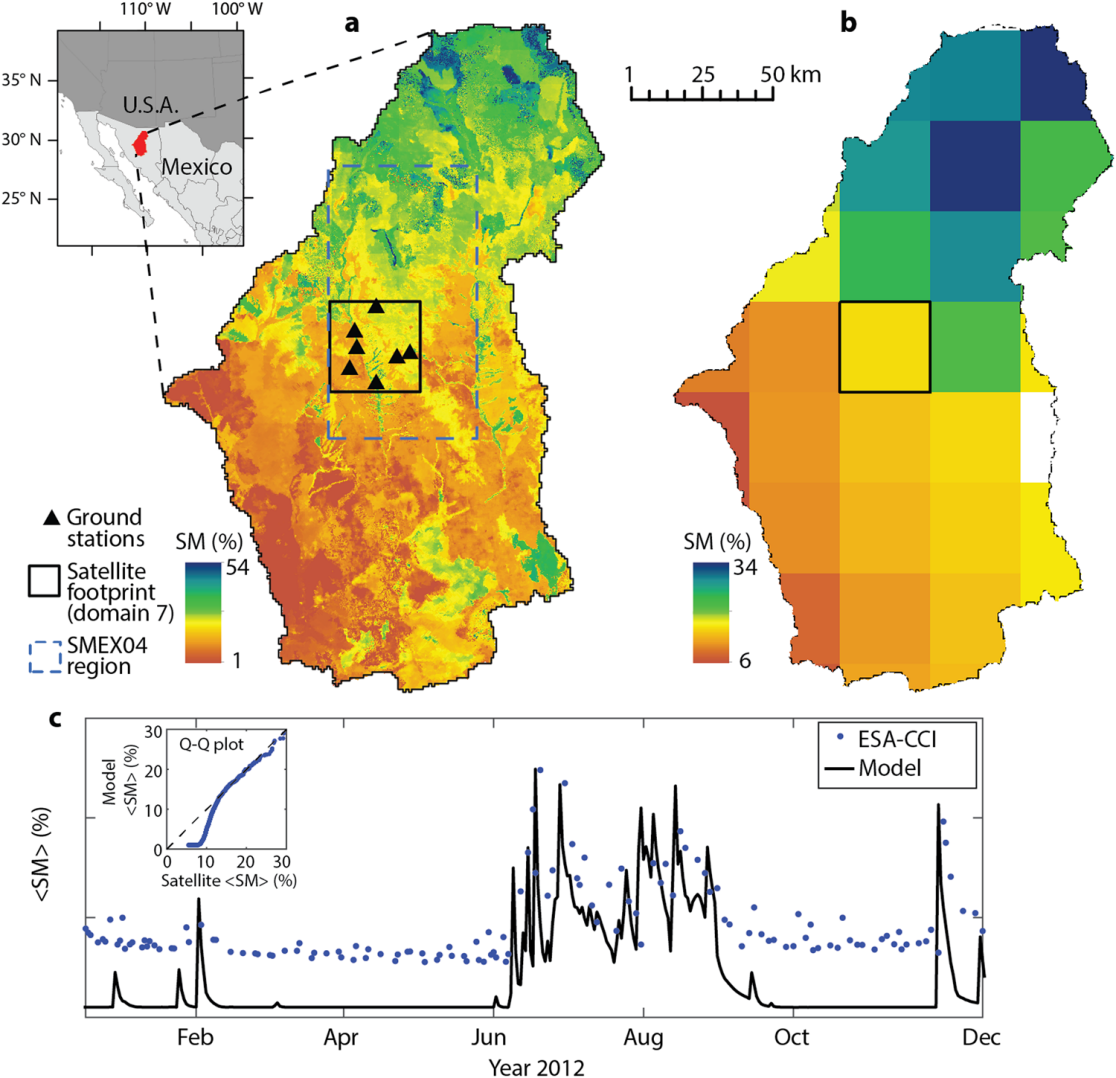
Analysis domain, hyperresolution simulations, and satellite data. (**a**) SM at 125-m resolution simulated by the hydrologic model on 16 July 2012 in the RSB, along with the location of ground stations, the 32 km × 32 km satellite footprint (domain 7), and the 75 km × 50 km SMEX04 area. (**b**) SM estimates derived by ESA-CCI in the RSB on 16 July 2012, regridded at 32-km. (**c**) Time series of the daily spatial mean SM, <*SM*>, in domain 7 simulated by the hydrologic model and retrieved by ESA-CCI for the year 2012. Inset: Q-Q plot between simulated <*SM*> and ESA-CCI retrievals in domain 7 from 2004 to 2013.

Figure 1 compares the simulated SM against ESA-CCI satellite products^11^ (see Methods). During the North American monsoon (NAM, July-September), SM varies significantly in space due to the interaction of localized storms, complex topography, and heterogeneities in soil and vegetation properties^30,31^. Simulations capture well this variability, as shown for a summer day in Fig. 1a, where SM exhibits both large-scale variability and fine-scale heterogeneity. The ESA-CCI products also capture the large-scale SM variability well, but inherently lack high-resolution information (Fig. 1b). Furthermore, the strong observed seasonality in precipitation and vegetation greenness impacts the temporal dynamics of SM as well. For instance, Fig. 1c presents the daily simulated and satellite-derived SM averaged within the satellite footprint of domain 7 for the year 2012 (see Methods). The satellite products capture very well the seasonal dynamics during the NAM season and all peaks (correlation coefficient of 0.6 and root mean square error of 6.6% during the simulation period), but overestimate SM in dry conditions as found for ESA-CCI retrievals at other sites^11^ (see Q-Q plot in the inset of Fig. 1c).

**Figure 2:**
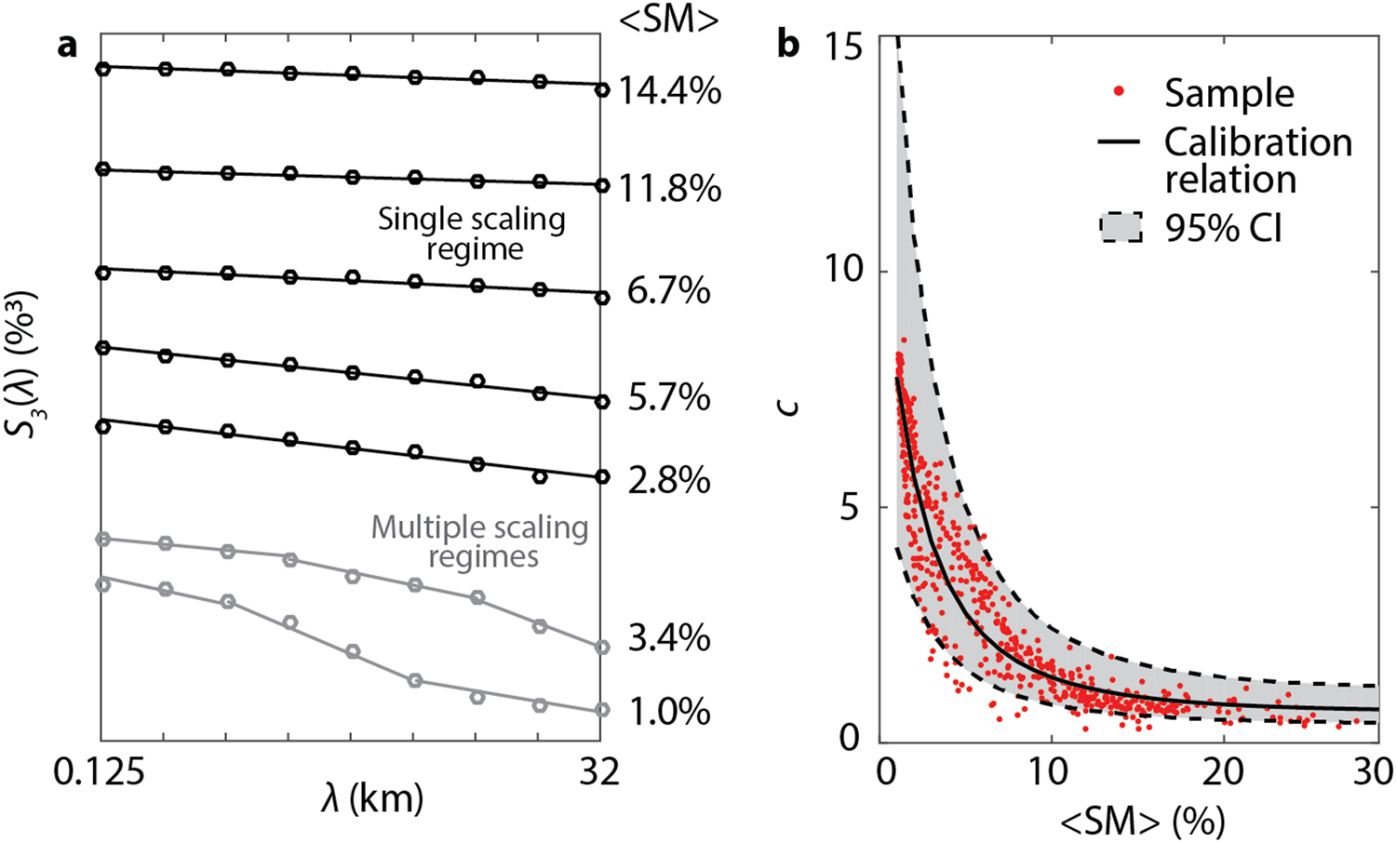
Scale invariance of simulated soil moisture and calibration of the downscaling algorithm. (**a**) Scale invariance analysis from *l* = 125 m to *L* = 32 km for a set of representative days of 2012, with evidence of a single (black) and multiple (gray) scaling regimes. The coarse-scale mean SM, <*SM*>, is reported for each day. (**b**) Calibration relation (continuous line) of the downscaling algorithm for domain 7 (Fig. 1a), estimated with data of 2005–2009. The 95% C.I. for future individual data values not used for the regression are plotted as shadings and dashed lines. Estimates of the parameter *c* for 2010–2013 are shown with red circles.

Scale invariance is investigated in the hyperresolution SM fields from the fine scale of 125 m to the coarse scale of 32 km over multiple domains in the basin that capture several ecosystems, including shrublands, desert scrub, oak and mesquite savannas, and pasture (Fig. S4). A single scaling regime is found across a large range of wetness conditions, as shown in Figs 2a and S1 by the log-log linearity between the third moment, *S*_3_(*λ*), and the scale, *λ*. Multiple scaling regimes are found (1) in very dry conditions when SM is close to the residual moisture content, and (2) when coarse precipitation data cause the presence of marked discontinuities in the spatial distribution of SM (see Fig. 2a and results for 19 September and 15 April 2012 in Fig. S1). Since these exceptions are most likely due to the coarse resolution of geospatial datasets and forcings used in the hydrologic simulations, we argue that multiple scaling regimes exhibited by the simulated fields do not reflect the actual statistical behavior of SM in these days. Overall, results of this analysis suggest that the scaling regime previously observed up to ~1 km through aircraft SM estimates can be extended to scales representative of field observations (~125 m).

### Soil moisture downscaling of satellite data

The hyperresolution SM fields are used to calibrate a downscaling algorithm that reproduces observed scale invariant properties through a stochastic generator of multifractal cascades^32^ (see Methods and Fig. S2). This parsimonious approach has ideal characteristics for operational use. Its two parameters, *c* and *β*, are derived through calibration relations with coarse-scale predictors and used to generate an ensemble of equally probable fields reproducing the small-scale probability density function (PDF) of SM. As in previous applications^13–15^, *β* is found to be constant in the basin, while *c* is linked to the coarse-scale SM, <*SM*>(see Methods). Figure 2b shows the calibration relation estimated for domain 7 using simulations from 2005 to 2009. The 95% confidence intervals (C.I.) of the regression are also plotted along with the *c* estimates for SM fields of 2010–2013. Only 6% of the *c* estimates are not included within the 95% C.I., a percent very close to the expected 5%, thus supporting the robustness of the estimated empirical relation. Similar results are found for the other domains (see Fig. S5).

The downscaling algorithm ability to capture the distribution of the hyperresolution SM fields is first tested. To do so, for each day and coarse-scale domain, we compute <*SM*> as the spatial mean of the hyperresolution SM products and generate an ensemble of 100 downscaled fields at 125-m resolution. In Fig. S1, we compare the PDFs of the hyperresolution SM in domain 7 for seven representative days of 2012, against the 95% C.I. derived from the downscaled fields. The downscaling algorithm is able to capture a large range of PDF shapes, with very good performances in dry and medium wetness conditions (9 February, 28 June, and 31 December) and good performances in the wettest days (16 July and 8 September). For the cases exhibiting multiple scaling regimes, performances are still adequate in very dry conditions where SM is close to the residual moisture content (15 April) and degrade when precipitation fields cause spatial discontinuities (19 September). To summarize the downscaling performance, Figs S3 and S6 show that the standard deviation of the hyperresolution SM distributions is reproduced remarkably well by the downscaled fields in the vast majority of days and domains. The skewness is also simulated with good accuracy, except for a few outliers (see Methods).

**Figure 3:**
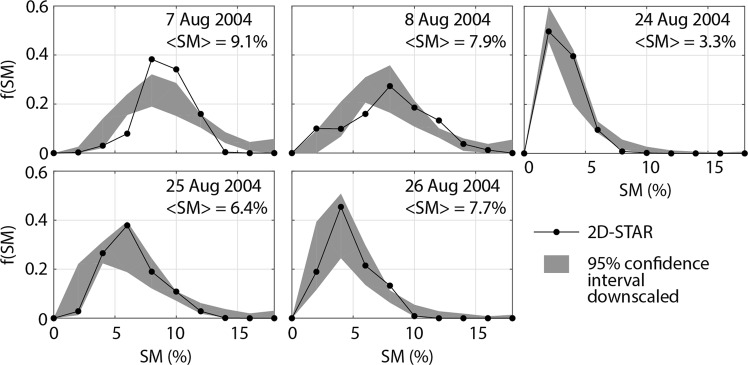
Downscaling algorithm performance against aircraft soil moisture estimates. Comparison between the PDFs of SM at 1000-m resolution retrieved by 2D-STAR during SMEX04 and the 95% C.I. derived from 100 downscaled fields.

The downscaling algorithm calibrated with the hyperresolution hydrologic simulations is then applied to simulate the small-scale distribution of two independent SM datasets available in domain 7. We first compare the downscaled fields against aircraft SM products retrieved by L-band 2-D Synthetic Aperture Radiometer (2D-STAR) observations during five days of the Soil Moisture Experiment 2004 (SMEX04) campaign held in August of 2004^33^ (the SMEX04 region is reported in Fig. 1a). To apply the downscaling algorithm, we use the spatial mean of the 2D-STAR SM estimates as <*SM*> and a fine scale of 1000 m, which is the closest to the aircraft footprint. As shown in Fig. 3, the downscaled SM distributions capture very well the PDFs of 2D-STAR SM, which include both skewed and symmetric shapes. The only exception is on 7 August when the downscaling algorithm overestimates the spatial variability of SM, which was relatively low due to the occurrence of a large storm on the previous day.

**Figure 4:**
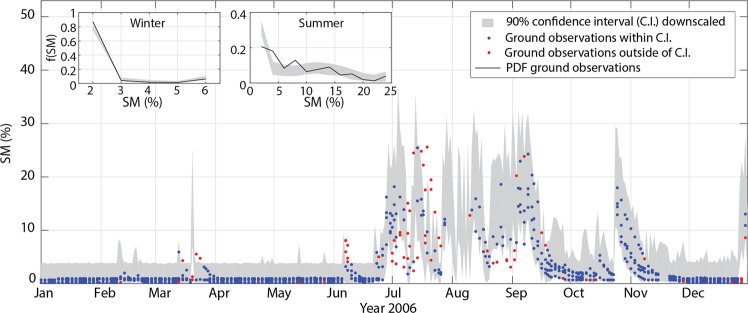
Downscaling algorithm performance against ground soil moisture observations. Comparison between ground observations of surface SM in 2006 and 90% C.I. of the spatial distribution of SM downscaled at 125-m resolution from the bias-corrected ESA-CCI SM products and, when these are not available, from the aggregated hyperresolution simulations. Insets show the PDFs of ground SM observations in winter (January-March and October-December) and summer (July-September) along with the corresponding 90% C.I.s derived from the downscaled values (see Methods).

We then apply the multifractal algorithm to downscale the ESA-CCI SM products up to 125 m and compare the downscaled fields against ground SM observations. For this effort, we select year 2006 because of the largest number of quality-controlled observations in domain 7 and only 11 days with missing data. To limit errors of the retrieval algorithm, we first bias correct the ESA-CCI SM estimates through a quantile mapping approach^34^ based on the Q-Q plot obtained from 2004 to 2013 (inset in Fig. 1c). For each day, we generate an ensemble of 100 downscaled fields. In days when satellite SM estimates are not available, we use the spatial mean of the hyperresolution simulations as the coarse-scale SM. Figure 4 shows the time series of the 90% C.I. of the small-scale SM distributions at 125-m resolution along with the ground observations. The spatiotemporal variability of ground SM data is captured extremely well across a wide range of wetness conditions, with 91% of the ground observations included within the 90% C.I. of the downscaled products. To our knowledge, this is the first demonstration of the downscaling of satellite SM data to the resolutions relevant to field conditions.

## Discussion

### Implications for modeling and operations at local scales

This study closes the loop of surface SM estimation at scales that are relevant for local applications (~100 m) by applying an efficient statistical procedure that uses information collected at coarse scales (~50 km) from Earth-orbiting satellites. These include the recent Soil Moisture Ocean Salinity^35^ and Soil Moisture Active and Passive^36^ missions that are dedicated to SM monitoring. High-resolution SM data generated by the downscaling algorithm can be used to support irrigation districts^9^ and forest fire operations^37^, and assimilated in predictive models to forecast convective thunderstorms^38^ and flash floods^39^. Furthermore, they are useful to improve the characterization of the nonlinear parameterizations of SM and land surface fluxes adopted in climate and weather models^1^. The calibration of the downscaling algorithm was only possible through the use of information on scale invariance obtained from a hydrologic model run at hyperresolution. While this approach was tested in a specific region, the methodology proposed can be applied globally. As a result, the increasing availability of hyperresolution hydrologic simulations^40^ will permit the downscaling algorithm to be applied at regional to continental scales in other climatic regions and ecosystems.

## Methods

### The Río Sonora basin

The RSB drains an area of 21,237 km^2^ in the state of Sonora in northwest Mexico that is part of the NAM region (Fig. 1a). This basin is characterized by complex topography with strong elevation gradients (> 2400 m) and spatially heterogeneous ecosystems, dominated by shrubland, desert scrub, oak and mesquite savannas, and pasture^30,31^. Reference ^31^ presents maps of terrain, soil, and land cover for the basin. Irrigated and rainfed agriculture is practiced in a relatively small fraction of the basin. Climate in the RSB is classified as arid to semiarid with annual mean temperatures (precipitation) ranging from 11 to 29 °C (350 to 750 mm) depending on the location. Two main seasons can be identified. Summer is dominated by the NAM from early July to the end of September, during which 40–80% of the annual precipitation falls mainly in form localized convective thunderstorms^41,42^. As a consequence of the combined availability of precipitation and solar radiation^43^, the majority of the ecosystems experience a dramatic greening that peaks in mid-July and decays in late September. The winter season extends from November to March and is dominated by dry conditions with senescent vegetation, which are interrupted by a few large-scale precipitation events^44^.

### Hyperresolution hydrologic simulations

Hydrologic simulations were conducted in the RSB using the parallelized version of the Triangulated Irregular Network (TIN)‐based Real‐time Integrated Basin Simulator (tRIBS) distributed hydrologic model^27–29^. tRIBS is a physics-based model that simulates the coupled water-energy balance in continuous fashion, capturing the spatial variability of land surface properties through a variable-resolution domain. The model represents the main hydrologic processes over a land surface, including rainfall interception and soil infiltration, unsaturated and saturated flow, runoff generation, evaporation from bare soil and wet canopy, plant transpiration, and hillslope and channel routing. The use of approximate solutions in the infiltration and routing schemes allows tRIBS to be computationally efficient while adequately capturing process physics^45^, whereas the variable-resolution domain and subbasin decomposition during parallelization provide the capacity of simulating regional basins over long time periods.

A detailed description of model setup and testing in the RSB is provided in a previous study^30^. The model was applied at a hyperresolution^23,46^ of ~88 m over a decadal period from 2004 to 2013, integrating ground and remotely-sensed observations. Hydrometeorological forcings were derived from the 12-km North American Land Data Assimilation System. Precipitation was bias corrected using daily rain gauge observations, while meteorological variables were downscaled at 1 km using terrain information. To capture vegetation seasonality, time-varying vegetation parameters were derived from the Moderate Resolution Imaging Spectroradiometer (MODIS) sensor at resolutions from 250 to 1000 m. High‐resolution (250‐m) grids of soil parameters were generated by integrating a coarse‐resolution soil map based on the Food and Agriculture Organization classification with recently released global data sets^30^. Soil parameters were calibrated against hourly SM observations of nine stations installed within each soil class. Model simulations were tested against: (i) independent SM observations at another 11 stations and for different time periods; and (ii) daily estimates of land surface temperature derived from MODIS at 1-km resolution. The hyperresolution simulations were found to capture well the surface (0–5 cm) SM temporal variability observed at distinct locations and the spatial variability of land surface temperature, a variable strongly related to SM. In this study, we use daily outputs of surface SM, resampled from the irregular mesh to regular grids at 125-m resolution (see Fig. 1a and panels on the first column of Fig. S1 for examples of these products).

### Ground and remotely-sensed soil moisture data sets

Ground observations of surface SM are obtained by a network of stations installed in the RSB, with the goal of conducting studies on the impacts of the NAM on regional ecosystems and hydrology^47^. A total of 20 stations were gradually installed from 2004 to 2013 over a large area covering the central and upper portions of the basin. Here, we use data of the seven stations located within the 32 km × 32 km domain reported in Fig. 1a. In addition, we use aircraft SM products retrieved by L-band 2-D Synthetic Aperture Radiometer (2D-STAR) observations during five days of the Soil Moisture Experiment 2004 (SMEX04) campaign held in August of 2004^33^. The SMEX04 region covers an area of 75 km × 50 km in the RSB that is shown in Fig. 1a. Aircraft SM products, available at 800 m resolution, are aggregated at 1000 m.

Satellite-based estimates of surface SM are obtained from the European Space Agency (ESA) Climate Change Initiative (CCI)^11^. The ESA-CCI SM estimates are generated by merging Level 2 SM retrievals from a set of C-band active sensors and multi-frequency passive sensors. The ESA-CCI algorithm combines multiple single SM retrievals. To account for the differences between products, a correction based on long-term SM estimates from the GLDAS-Noah v1 land surface model^48^ is applied^49,50^. Here, we use the ESA-CCI v04.4 COMBINED SM products, available from 1 November 1978 to 6 June 2018. While provided daily in a 0.25° grid, the actual resolution varies in space and time because: (i) the Level 2 SM input products have different resolutions ranging from 25 to 50 km and revisit times of 1 to 2 days; and (ii) retrievals from different sets of active and passive sensors are merged at different times, depending on their availability. The accuracy of the ESA-CCI products is affected by a number of factors, including instrument type, position of the satellite with respect to the observed area, local surface conditions, and radio frequency interference, among others. In our study region, the main factors that can negatively impact the SM retrievals are complex terrain, the presence of vegetation during the NAM season, and the presence of dry soils that can cause scattering effects^11^.

### Scale invariance and multifractal analysis of soil moisture

We carry out scale invariance and multifractal analyses on simulated SM following an approach based on binary cascades^13^. These analyses are conducted from a coarse scale, *L*, to a fine scale, *l*, related as $L=l\cdot {2}^{{N}_{lev}}$, where *N*_*lev*_ are the aggregation levels. The first step is the computation of the structure function *S*_*q*_(*λ*) at a given scale *l* ≤ *λ* ≤ *L* and for the moment *q*:

1 $${S}_{q}(\lambda )=\frac{1}{N{(\lambda )}^{2}}{\sum }_{i=1}^{N(\lambda )}{\sum }_{j=1}^{N(\lambda )}{[S{M}_{i,j}(\lambda )]}^{q},$$

where *SM*_*i*,*j*_(*λ*) is the SM at scale *λ* in the pixel (*i*, *j*), and $N{(\lambda )}^{2}={(\frac{L}{\lambda })}^{2}$ is the number of *λ* × *λ* pixels in the coarse domain *L* × *L*. Scale invariance is found if a power law holds:

2 $${S}_{q}(\lambda ) \sim {\lambda }^{-K(q)},$$

where *K*(*q*) is called multifractal exponent. This is done in practice by testing the linearity of the log-log transformation of Eq. (2), where *K*(*q*) is estimated as the slope of the line. The presence of the relation (2) is tested for different values of the moments *q*. The spatial field is defined fractal, if the relation between *K*(*q*) and *q* is linear, or multifractal, if it is nonlinear.

We assume a coarse scale *L* = 32 km to mimic the footprint resolution of most satellite products, and a fine scale *l* = 125 m of the simulated SM fields. We investigate Eq. (2) for *q* = 1.5, 2, 2.5, 3, and 3.5. Figure S1 shows examples of the presence of scale invariance and multifractality in the coarse domain identified in Fig. 1a,b (domain 7). Analyses are also carried out in other 15 non-overlapping 32 km × 32 km domains spanning the entire RSB (Fig. S4). Overall, we find scale invariance to be present from 125 m to 32 km across a large range of wetness conditions, with a few exceptions where the relation between the logarithmically transformed *S*_*q*_(*λ*) and *λ* does not exhibit a clear linear behavior. To exclude these cases from the calibration of the downscaling algorithm, we calculate the root mean square error (RMSE) between *S*_3_(*λ*) and the regression line, and disregard the days when RMSE is larger than a threshold of 0.12, determined empirically (e.g., 15 April and 19 September 2012 in Fig. S1).

### Soil moisture downscaling algorithm

Scale invariance analyses are used to apply a multifractal downscaling algorithm previously tested using aircraft-based surface SM products across Unites States and Australia^12–15^. A schematic of the downscaling approach is presented in Fig. S2. The algorithm reproduces observed SM properties through a log-Poisson stochastic generator of homogeneous random binary cascades. The generator provides an analytical expression for the multifractal exponent *K*(*q*) as a function of two parameters, *c* and *β*:

3 $$K(q)=c\cdot \frac{q(1-\beta )-(1-{\beta }^{q})}{\mathrm{ln}\,2}.$$

Parameters *c* and *β* are estimated on each analyzed SM field by fitting (3) to the sample multifractal exponents. Empirical calibration relations are then found between the set of *c* and *β* values and coarse-scale predictors, which allow the downscaling algorithm to be operationally applied. Here, we find a constant *β* = 0.89 to be appropriate; this value is within the range 0.71–0.96 found in other climatic regimes^12–15^. We then link *c* with the mean SM in the coarse-scale domain, <*SM*>, through a negative exponential equation:

4 $$y={y}_{\infty }+a\cdot exp(-\gamma  < SM > ),$$

where *y* = *c*^−0.1^ is a power transformation of *c*, and *y*_∞_, *α*, *γ*, are parameters. We apply a power transformation to *c* to improve the regression and guarantee the homoscedasticity of the residuals. Empirical calibration relations in the 16 domains are shown in Fig. S5.

### Downscaling algorithm validation

The application of the downscaling algorithm involves the following steps: (1) for a given coarse-scale SM value, <*SM*>, Eq. (4) is used to derive the expected value of *c*, *μ*_*c*_, and its standard deviation, *σ*_*c*_, estimated as half the width of the 95% C.I. of the regression line; (2) a set of *N* values of *c* are randomly generated from a normal distribution with mean *μ*_*c*_ and standard deviation *σ*_*c*_; and (3) this set of *c* values are used to generate *N* downscaled fields at 125-m resolution with the downscaling algorithm. For all our verifications, we assume *N* = 100. We test the downscaling algorithm against: (i) SM fields simulated by the hydrologic model (Figs S1, S3, and S6); (ii) 2D-STAR aircraft SM products (Fig. 3); and (iii) ground SM data (Fig. 4). In all cases, we use the calibration relations estimated with the 2005–2009 SM outputs. Comparisons between the reference high-resolution SM fields and the 90% and 95% C.I. derived from the downscaled fields are made through PDFs (Figs 3, 4 and S1), standard deviation and skewness (Figs S3 and S6), and range of the empirical distribution (Fig. 4). For the comparison with the PDFs of ground observations shown in the insets of Fig. 4, the 90% C.I. are obtained from an ensemble of downscaled distributions with the sample size of the available observations. Each of these distributions is built by randomly extracting, in each day, a number of downscaled values equal to the available observations and, then, pooling the values for all days together.

## Supplementary information

Supplementary Information

## Data Availability

The datasets generated during and/or analyzed during the current study are available from the corresponding author on reasonable request.
