# Supplementary material for: Closing the Loop of Satellite Soil Moisture Estimation via Scale Invariance of Hydrologic Simulations
Source: Sci Rep. 2019 Nov 6;9:16123. doi: 10.1038/s41598-019-52650-3 (PMC6834674; doi:10.1038/s41598-019-52650-3)
Supplement: Supplementary file 1 — Supplementary Information [file 41598_2019_52650_MOESM1_ESM.pdf]

Closing the Loop of Satellite Soil Moisture Estimation via Scale Invariance of Hydrologic Simulations

Giuseppe Mascaro[1], Ara Ko[1], and Enrique R. Vivoni[1,2]

[1] School of Sustainable Engineering and the Built Environment, Arizona State University, Tempe, AZ, USA

[2] School of Earth and Space Exploration, Arizona State University, Tempe, AZ, USA.

Supplementary Information

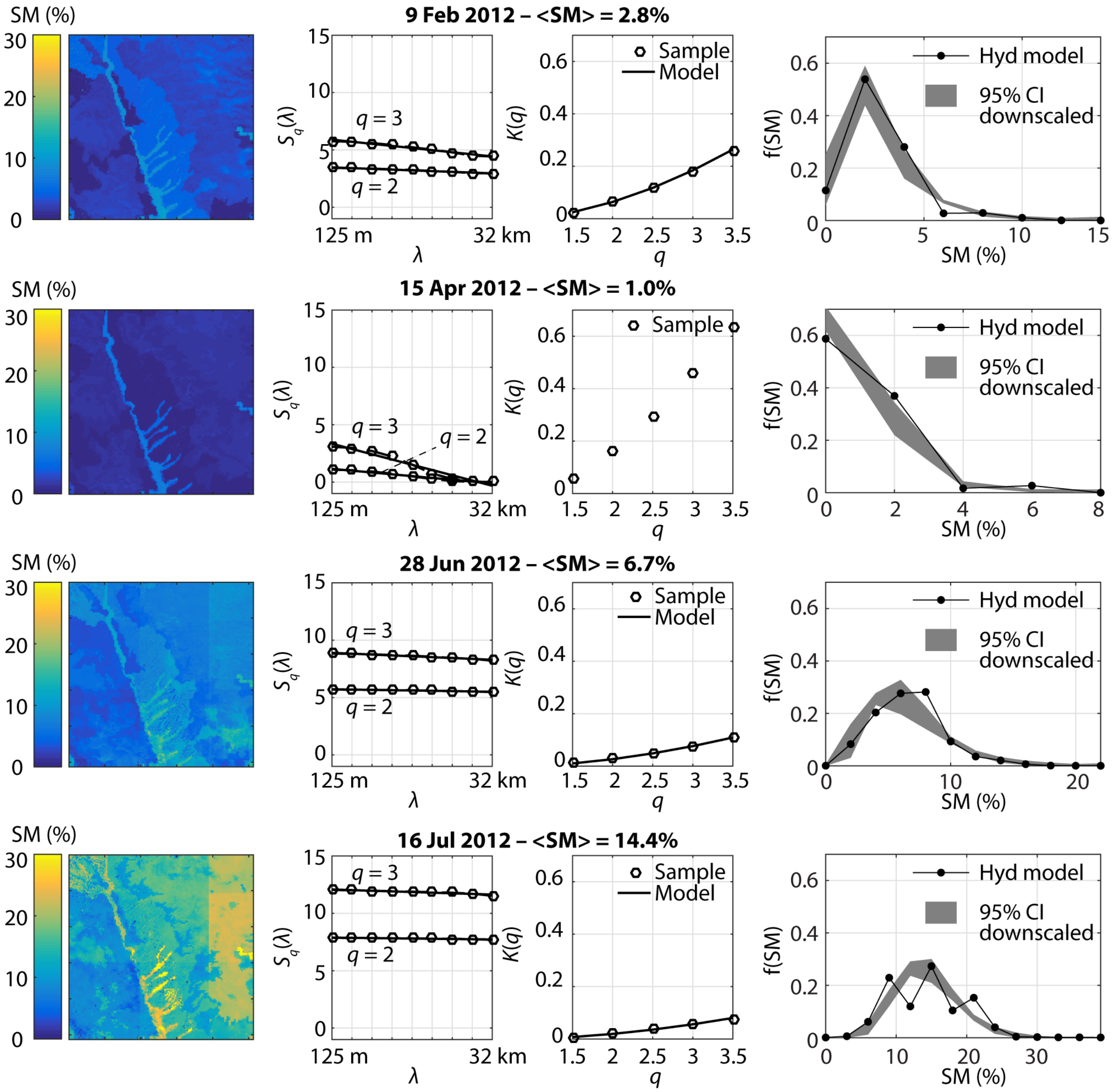

Figure S1. First column: surface soil moisture (SM) fields simulated by the tRIBS hydrologic model at 125-m resolution in the 32 km x 32 km domain identified in Figs. 1a,b (domain 7). Second column: scale invariance analysis showing the relation between $S_q(\lambda)$ and λ for $q = 2$ and 3 in the log-log space; $S_q(\lambda)$ values are shown with circles, while the regressions lines are plotted in black. Third column: multifractal analysis showing the relation between the sample multifractal exponents $K(q)$ and the moments q, along with the theoretical expectation of the

log-Poisson of equation (3) (model); the latter is not reported when the root mean square error between $S_3(\lambda)$ and the regression line is larger than 0.12. Fourth column: comparison between (i) the probability density function (PDF) of SM at 125-m resolution simulated by the hydrologic model, and (ii) the 95% confidence intervals derived from 100 downscaled fields. Results are shown for a set of representative days of year 2012 spanning a range of coarse-scale mean SM, $<SM>$.

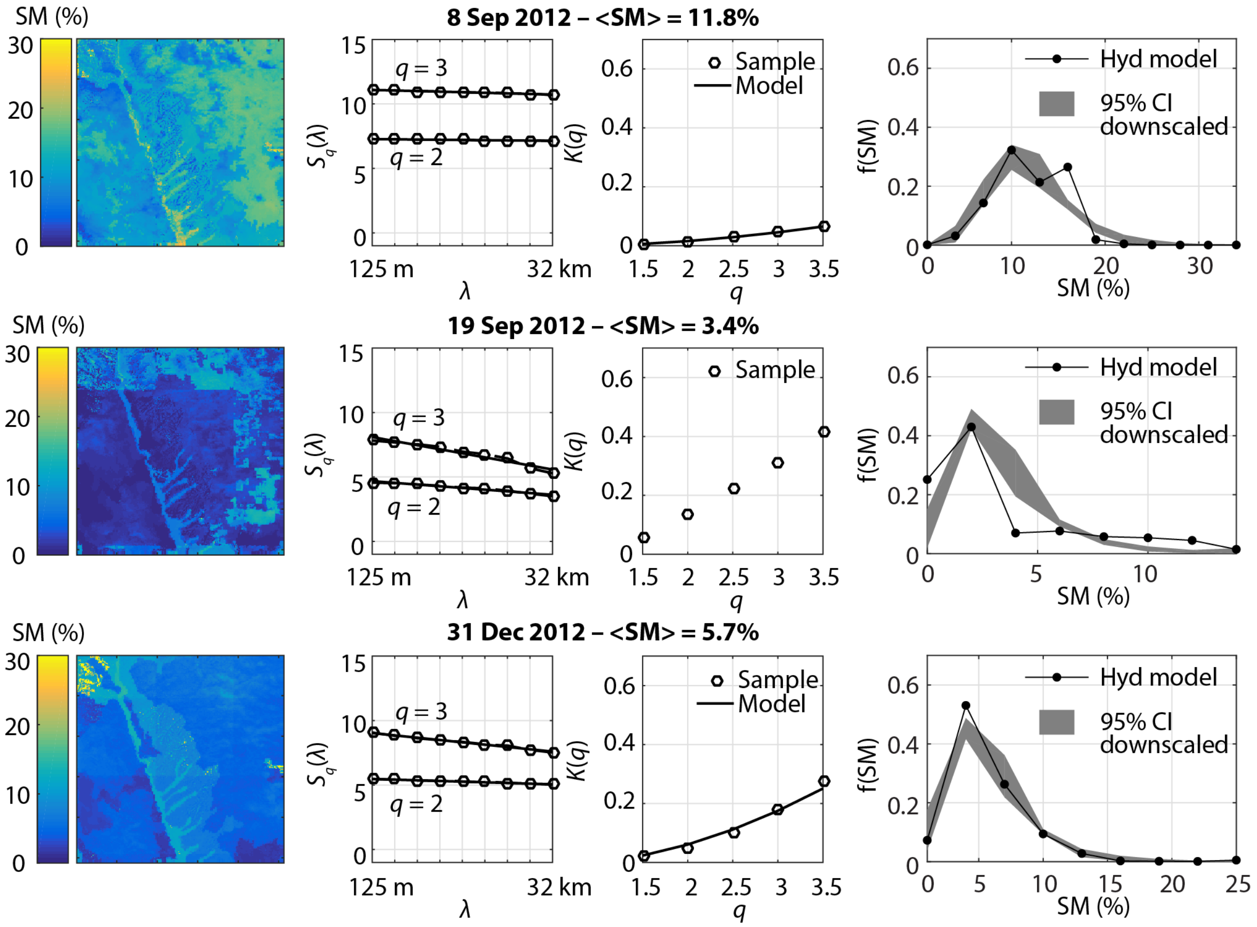

Figure S1. Continued.

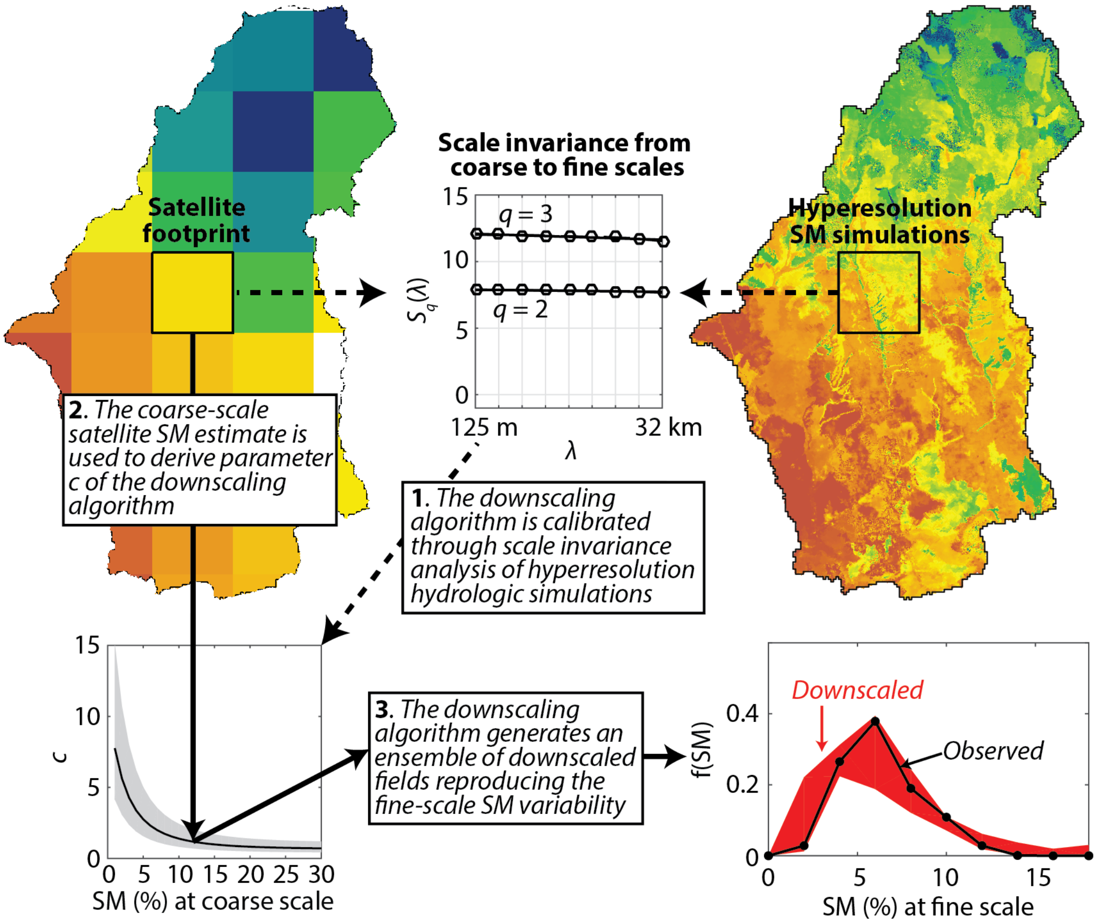

Figure S2. Flowchart describing the downscaling algorithm calibration (dashed lines; step 1) and application (solid lines; steps 2 and 3).

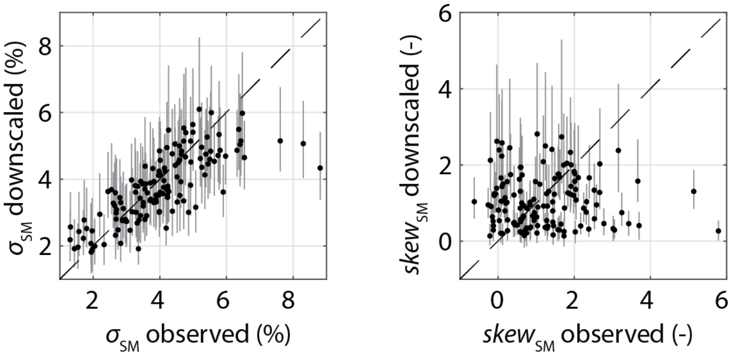

Figure S3. (a) Scatterplot of observed standard deviation, σ_{SM}, of 140 randomly selected SM fields simulated in domain 7 versus the ensemble average and 95% confidence intervals derived from 100 synthetic fields. (b) Same as (a) but for the skewness, $skew_{SM}$.

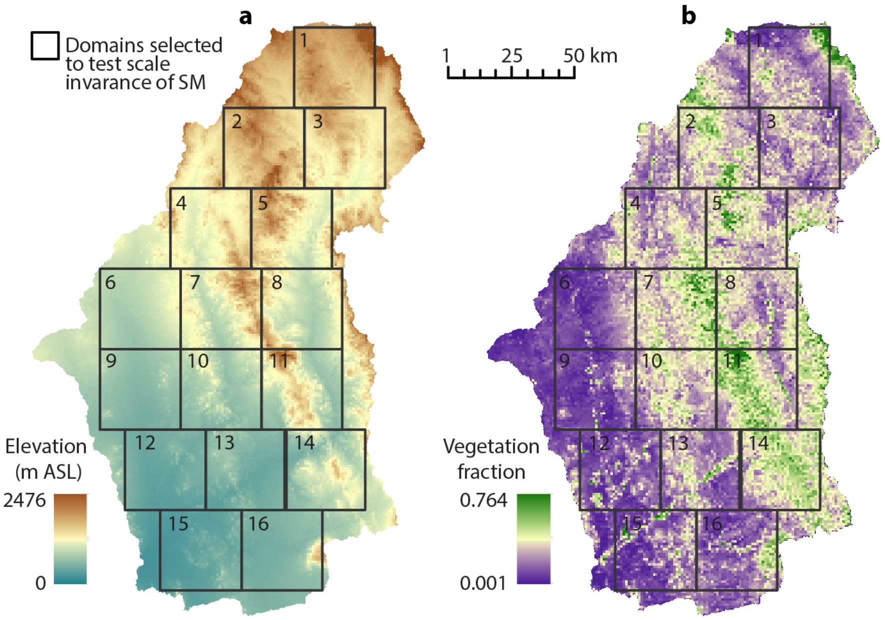

Figure S4. Coarse-scale 32 km x 32 km domains selected to investigate the presence of scale invariance in the Río Sonora basin, shown in the maps of (a) terrain and (b) mean annual vegetation fraction.

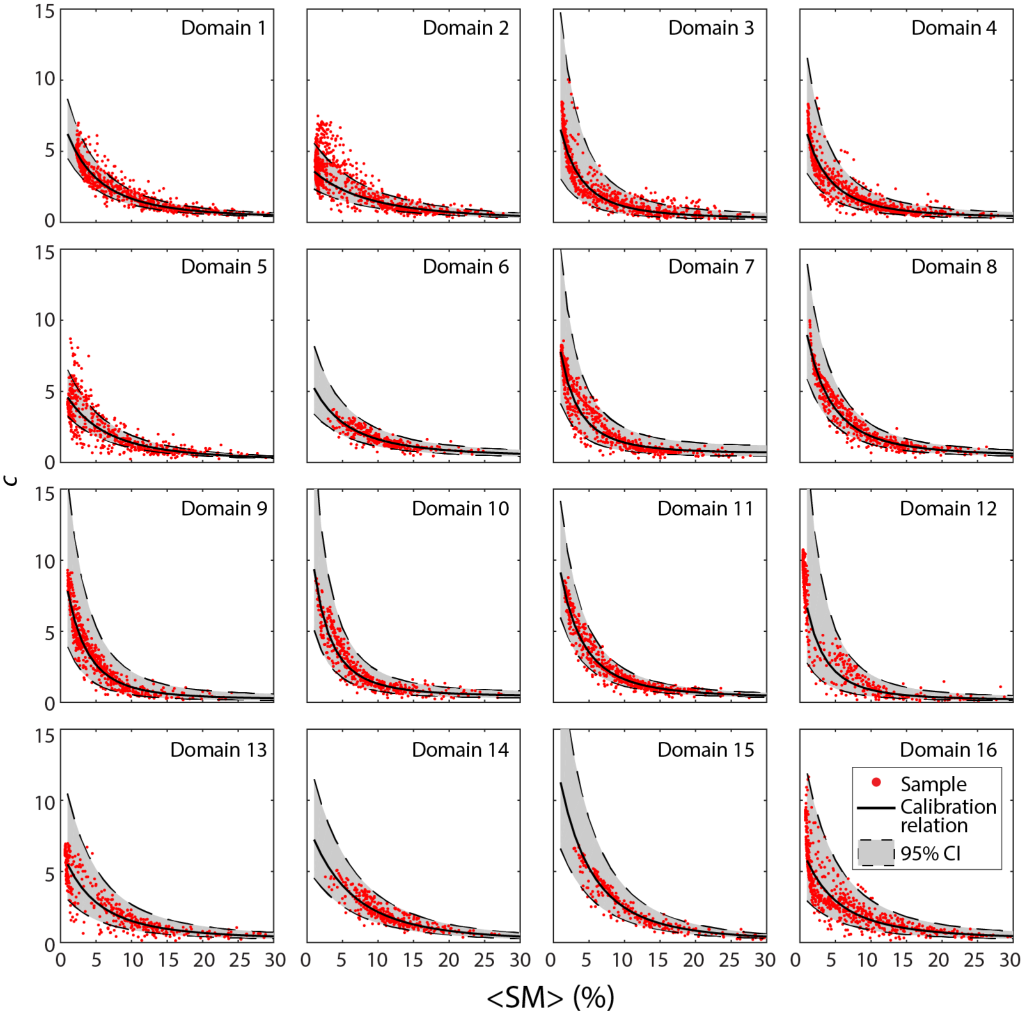

Figure S5. Same as Fig. 2b, but for the domains of Fig. S4.

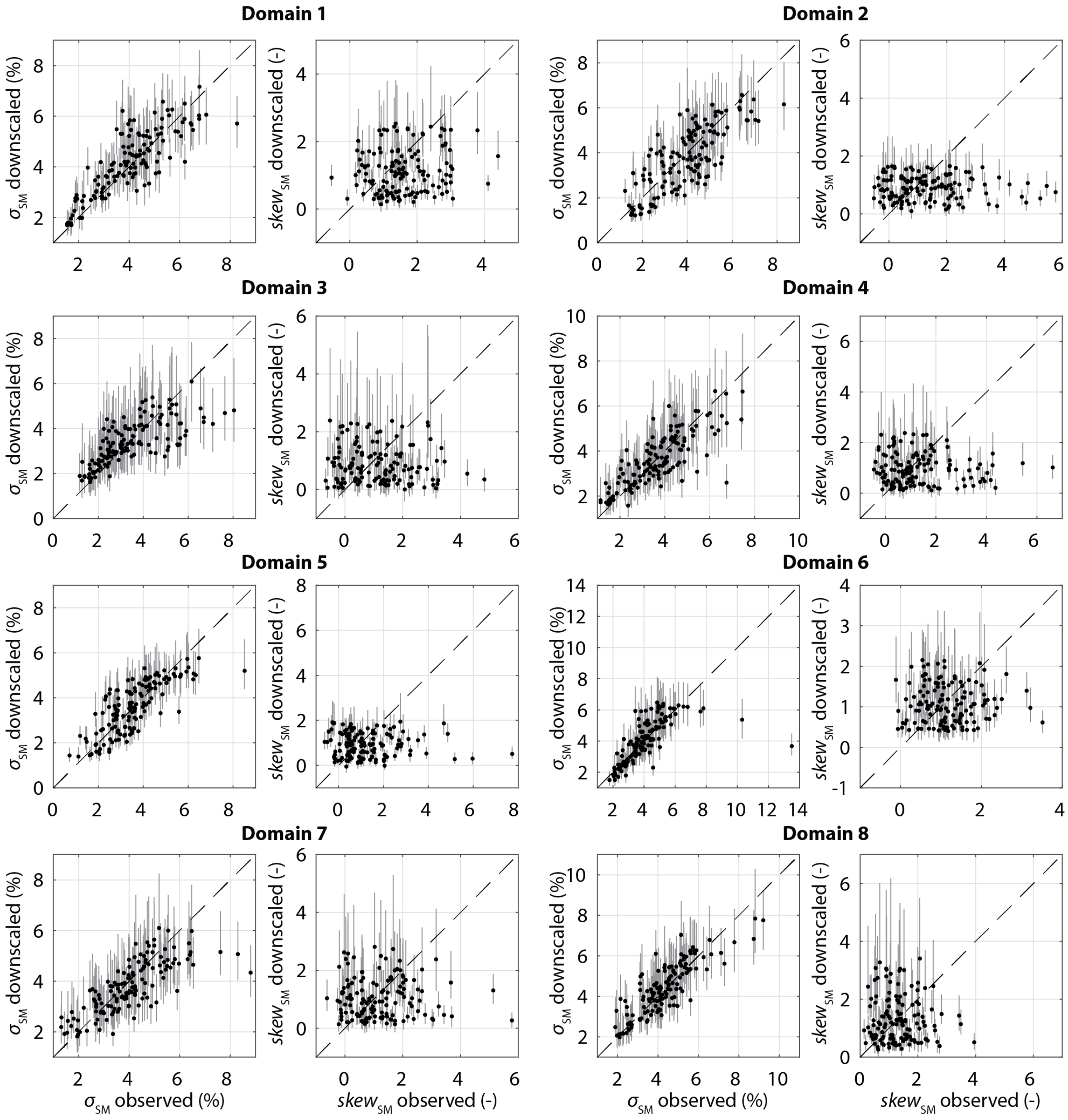

Figure S6. Same as Fig. S3, but for the domains of Fig. S4.

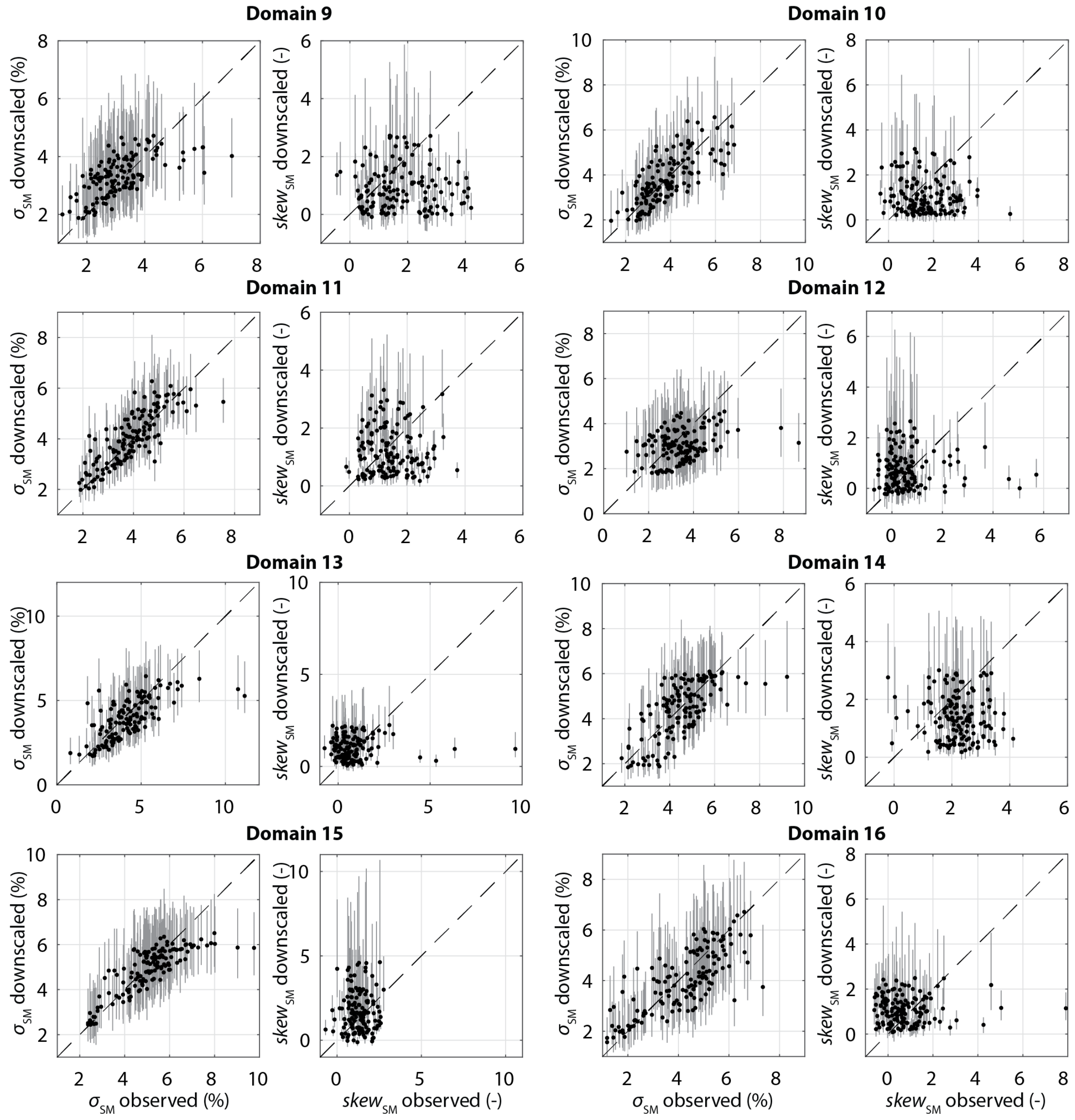

Figure S6. Continued.
